# A comprehensive approach for osteoporosis detection through chest CT analysis and bone turnover markers: harnessing radiomics and deep learning techniques

**DOI:** 10.3389/fendo.2024.1296047

**Published:** 2024-06-04

**Authors:** Kaibin Fang, Xiaoling Zheng, Xiaocong Lin, Zhangsheng Dai

**Affiliations:** ^1^ Department of Orthopaedic Surgery, The Second Affiliated Hospital of Fujian Medical University, Quanzhou, China; ^2^ Aviation College, Liming Vocational University, Quanzhou, China

**Keywords:** deep learning, osteoporosis, computed tomography, radiomics, transfer learning

## Abstract

**Purpose:**

The main objective of this study is to assess the possibility of using radiomics, deep learning, and transfer learning methods for the analysis of chest CT scans. An additional aim is to combine these techniques with bone turnover markers to identify and screen for osteoporosis in patients.

**Method:**

A total of 488 patients who had undergone chest CT and bone turnover marker testing, and had known bone mineral density, were included in this study. ITK-SNAP software was used to delineate regions of interest, while radiomics features were extracted using Python. Multiple 2D and 3D deep learning models were trained to identify these regions of interest. The effectiveness of these techniques in screening for osteoporosis in patients was compared.

**Result:**

Clinical models based on gender, age, and β-cross achieved an accuracy of 0.698 and an AUC of 0.665. Radiomics models, which utilized 14 selected radiomics features, achieved a maximum accuracy of 0.750 and an AUC of 0.739. The test group yielded promising results: the 2D Deep Learning model achieved an accuracy of 0.812 and an AUC of 0.855, while the 3D Deep Learning model performed even better with an accuracy of 0.854 and an AUC of 0.906. Similarly, the 2D Transfer Learning model achieved an accuracy of 0.854 and an AUC of 0.880, whereas the 3D Transfer Learning model exhibited an accuracy of 0.740 and an AUC of 0.737. Overall, the application of 3D deep learning and 2D transfer learning techniques on chest CT scans showed excellent screening performance in the context of osteoporosis.

**Conclusion:**

Bone turnover markers may not be necessary for osteoporosis screening, as 3D deep learning and 2D transfer learning techniques utilizing chest CT scans proved to be equally effective alternatives.

## Background

Bone turnover markers (BTM) are biochemical substances produced during the dynamic process of bone remodeling, providing a timely and accurate reflection of bone turnover in the human body (1). These markers play a pivotal role in the diagnosis and treatment of osteoporosis, a prevalent bone disorder (2). However, the diagnosis of osteoporosis primarily relies on DXA (3). Although there is some correlation between BTM and BMD, this correlation is not robust enough for the diagnosis of osteoporosis (4). These limitations have hindered the widespread use of these markers. The diagnosis of osteoporosis currently requires quantitative CT or DXA examinations, which may increase additional costs (5). Furthermore, the availability of these devices is limited, particularly QCT, in many medical centers. Simultaneously, it is worth noting that the screening rate for osteoporosis remains unsatisfactory, likely indicating a lack of comprehensive understanding regarding the intricacies of the disease (6).

Chest CT is a crucial and commonly performed medical check-up. Regular chest CT scans are recommended for certain populations, particularly the elderly, who are considered to be at higher risk for lung cancer (7). If this examination can successfully diagnose osteoporosis, it may potentially eliminate the need for DXA scans, thereby reducing radiation exposure.The technologies of deep learning and radiomics provide possibilities for the implementation of this idea. Radiomics refers to the extraction of data that can be analyzed from medical imaging, and it has been extensively applied in enhancing the accuracy of medical diagnosis, prognosis, and clinical decision-making. Its application aims to achieve precise medical treatment (8). This technology has gained widespread adoption and its efficacy has been validated (9, 10). Deep learning is also extensively utilized in the field of medicine. This technology is not only applied for disease diagnosis but also widely employed for the automatic segmentation of medical images (11). This technology has also been employed in the diagnosis of osteoporosis and has yielded promising outcomes. Previously reported studies primarily focused on analyzing 2D images such as lumbar and hip X-rays, using deep learning techniques to diagnose osteoporosis in patients (12). However, in medical imaging, three-dimensional images such as CT scans and MRI scans are more commonly used. In this regard, employing pre-trained 3D deep learning models can significantly enhance the analysis of such medical images.

In this study, we aim to develop a comprehensive screening model for osteoporosis by integrating patient demographics and bone turnover markers.We also utilize radiomics techniques and both 2D and 3D deep learning algorithms to analyze chest CT scans and identify potential cases of osteoporosis. To extract transfer learning, transfer learning will be employed. Transfer learning enables the acquisition of valuable features from a source domain, encoding these features, and transferring them from the source domain to the target domain, thus effectively enhancing the performance of the target domain task (13).

This study aims to identify the most optimal methods for osteoporosis screening utilizing chest CT scans. It will explore and compare various techniques including radiomics, 2D and 3D deep learning, and 2D and 3D transfer learning techniques. Additionally, these methods will be compared to conventional bone turnover markers for their efficacy in osteoporosis screening.

## Materials and methods

### Participants in the study and development of clinical models

This study retrospectively analyzed a population of patients who underwent both chest CT scans and DXA bone density testing at a large hospital from January 2019 to May 2023. Patients with the following conditions will be excluded from the scope of the study: Severe scoliosis, both the eleventh and twelfth thoracic vertebrae with severe compressibility fractures that cannot be corrected, the fixed artifacts affecting the feature extraction area, and no results of bone turnover marker examination. Approval was obtained from the Hospital Institutional Review Board, and the study was conducted in compliance with the principles outlined in the Declaration of Helsinki. Almost all patients underwent chest CT scans and bone metabolism marker detection. The BTM included in the analysis were vitamin D, total type 1 collagen amino acid extension peptide (TPINP), and β- B-Cross Laps. The gold standard for distinguishing osteoporosis was the result of DXA, whereby a T-value of -2.5 or less indicated the presence of the condition (12).

The patients were randomly assigned to training sets, and their baseline data is depicted in **Table 1**. The clinical characteristics of the patients were analyzed using either an independent sample t-test or chi-square test, depending on the type of data.

**Table 1:** The baseline clinical characteristics of patients.

feature	train-no osteoporosis (n=170)	train- osteoporosis (n=222)	p_value	test-label=no osteoporosis (n=40)	test- osteoporosis (n=56)	p_value
Age(year)	62.97 ± 10.19	74.98 ± 11.55	<0.01	62.90 ± 13.34	68.61 ± 11.08	0.03
vitamin D (ng//ml)	27.08 ± 9.83	23.84 ± 10.01	<0.01	25.17 ± 7.81	26.28 ± 8.72	0.52
TPINP (ng//ml)	60.31 ± 43.55	70.52 ± 55.61	0.05	53.74 ± 38.81	67.56 ± 52.09	0.16
β-CTX (ng//ml)	0.48 ± 0.27	0.69 ± 0.45	<0.01	0.53 ± 0.28	0.58 ± 0.33	0.44
gender			0.01			0.16
male	65	51		12	10	
female	105	171		28	46	

TPINP, Total type I collagen amino terminal extender peptide.

β-CTX, β- Cross Laps.

### Clinical signature

The training set data underwent initial univariate analysis to identify factors with a p-value less than 0.05, indicating their significance. These selected clinical factors were then used for subsequent multivariate analysis.

To establish a predictive model, the eleven most common machine learning models were trained using the final selected clinical factors. These machine models include SVM (13), KNN (14), RandomForest (15), ExtraTrees (16), XGBoost (17), LightGBM (18), NaiveBayes (19), AdaBoost (20), GradientBoosting (21), LR (22), MLP (23).

### Delineation of ROI

**Figure 1:**
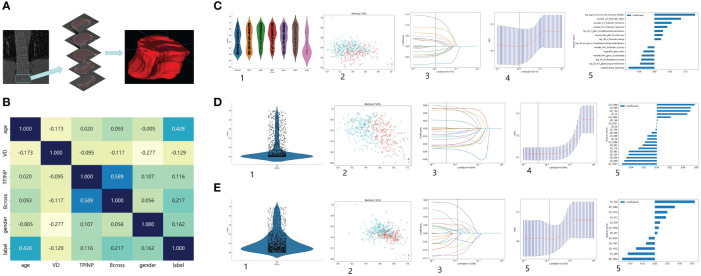
Research Process. **(A)** The process of outlining ROI. **(B)** The correlation between various clinical indicators and osteoporosis. **(C)** Extract Radiomics Features: 1. Overview of Extracted Features. 2. Visualize Results using Spectral Clustering. 3,4. LASOO Regression. 5. Feature Weights. **(D)** Extract 2D Transfer Learning: 1. Overview of Extracted Features. 2. Visualize Results using Spectral Clustering. 3,4. LASOO Regression. 5. Feature Weights. **(E)** Extract 3D Transfer Learning: 1. Overview of Extracted Features. 2. Visualize Results using Spectral Clustering. 3,4. LASOO Regression. 5. Feature Weights.

The preprocessing step involved adjusting the window width and window level of the images to bone windows, as well as standardizing the resolution of the images. Furthermore, for the purpose of standardization, all images will undergo adjustment to ensure consistent layer thickness and spacing. Radiomics feature extraction primarily focused on the twelfth thoracic vertebra during the analysis. In cases where measurement difficulties were encountered, the eleventh thoracic vertebra was selected instead to minimize potential deviations. This approach considered the physical susceptibility of the chest and waist, which are common areas for osteoporotic fractures resulting from movement and pressure (24). Axial images from the chest CT scans were chosen for analysis, and image reconstruction and delineation of the region of interest (ROI) were performed using ITK SNAP software (25). Typically, the images captured both vertebral bodies in the chest CT scans. Anatomical markers, such as the twelfth rib, the lower edge of the scapula, and the seventh cervical spine spinous process, were used to outline the segment of the thoracic spine for ROI delineation. The process of ROI drawing is illustrated in **Figure 1A**.

### Intra- and inter-observer variability

The intra- and inter-observer variability of the ROI delineation on the CT images was evaluated using the Intraclass correlation coefficient (ICC). One researcher defined the ROI, while another researcher with more than 10 years of experience in orthopedics randomly selected 30 cases and redefined the ROI. Both researchers were unaware of each other’s results. The ICC values were calculated based on these 30 cases. Prior to feature selection, an assessment of intra-observer variability will be performed on the extracted radiomics features of all patients. Features exhibiting intraclass correlation coefficients (ICCs) exceeding 0.9 will be deemed reliable and will proceed to subsequent analyses.

### Radiomics feature extraction

The feature extraction process was conducted using the Pyradiomics Module (https://github.com/Radiomics/pyradiomics).

### Feature selection

In order to identify the most relevant features associated with the presence of osteoporosis, a meticulous feature selection process was implemented. Initially, the U test (p<0.05) was employed to identify features that exhibited significant differences between the osteoporosis and non-osteoporosis groups. Furthermore, to ensure the inclusion of only statistically significant and reliable features, those with ICC coefficients lower than 0.9 were excluded from this step. This rigorous approach effectively reduced the number of features while maintaining their predictive power.To address the issue of multicollinearity, Pearson correlation analysis was conducted to examine the relationships between features. Calculation of correlation coefficients allowed for identification of feature pairs with values ≥0.9 or ≤-0.9. In such cases, only the feature demonstrating superior diagnostic performance was retained, thereby preventing redundancy within the model introduced by highly correlated features. Employing the Maximum Correlation Minimum Redundancy (mRMR) algorithm for feature selection, we retain only the top 20 most informative features. To further refine the feature set, the least absolute shrinkage and selection operator (LASSO) logistic regression technique was employed. We employed the same machine learning model used for analyzing clinical features to analyze the extracted radiomics features.

### 2D deep learning

The maximum cross-sectional area of ROI should be selected at first, as it represents the most prominent area of the thoracic vertebral body. These areas can then be cropped from the original CT image using Python. The source code for the cropping process is available open source and can be obtained from the CSDN website (https://blog.csdn.net/).

In this study, a pre-trained model was employed, and the researchers made no alterations to its parameters. Consequently, the study lacked a validation group, comprising solely a training group and a testing group (26). The division of this group aligns with the approach used in prior studies involving clinical and radiomics models.

The 24 most common deep learning neural network architectures are used for learning and recognizing images of these patients. These models are alexnet, densenet121, densenet169, googlenet, mnasnet1_0, mobilenet_v2, mobilenet_v3_large, mobilenet_v3_small, resnet101, resnet152, resnet18, resnet34, resnet50, resnext50_32x4d, squeezenet1_0, squeezenet1_1, vgg11, vgg11_bn, vgg13, vgg13_bn, vgg16, vgg16_bn, vgg19, vgg19_bn.

The cropped image serves as the input for deep learning algorithms. To update the model parameters, the stochastic gradient descent (SGD) optimizer is utilized. The training process consists of 100 epochs, each containing 1800 iterations. A batch size of 32 is used during these iterations. Each slice of the cropped image is treated as an independent input for the deep learning model.

### 3D deep learning

The complete Region of Interest (ROI) is extracted and serves as both the training and testing dataset for the 3D deep learning model. To update the model parameters, the stochastic gradient descent (SGD) optimizer is utilized. The training process consists of 100 epochs, each containing 1800 iterations. A batch size of 4 is used during these iterations. Each slice of the cropped image is treated as an independent input for the deep learning model. The 8 most common deep learning neural network architectures are used for learning and recognizing images of these patients. These models are denseNet121, resnet10, resnet101, resnet152, resnet18, resnet34, resnet50, shuffleNet. The parameter settings for 3D deep learning mirror those utilized in 2D deep learning.

### Transfer learning extraction

After completing both 2D and 3D deep learning, the most efficient deep learning model will be chosen for feature extraction. Once the feature extraction is finalized, these features will undergo a screening process identical to that used for radiomics features. Additionally, the same machine learning models would be employed for training and testing these features.

### Statistical analysis

The study will assess the efficacy of osteoporosis screening through a comparative analysis of radiomics models, deep learning models, transfer learning models, and clinical models. Ultimately, we will identify the model that demonstrates the highest screening efficiency.

To evaluate the performance of the model, data from the test set will be used. The effectiveness of the model will be assessed using the Area Under Curve (AUC) (27), a commonly employed metric in evaluating the performance of predictive models. The AUC provides a comprehensive measure of the model’s discriminatory ability and will be used to determine the overall quality of the predictions made by the model.

The patient’s baseline data were analyzed using statistical software packages, specifically SPSS (version 20.0) and Python. Continuous variables were presented as mean ± standard deviation, while categorical variables were described using frequencies and percentages. To assess the distribution of continuous variables, the Kolmogorov-Smirnov (KS) (28) test was employed. Additionally, the Levene test (29) was used to evaluate the homogeneity of continuous variances. To compare inter-group differences, the or Student’s t-test was used, depending on the distribution of the variables. For categorical variables, the Chi-squared test or Fisher’s exact test was employed. Statistical significance was defined as a p-value < 0.05. The AUC was used to evaluate the performance of predictive models, and the 95% confidence interval (CI) of the AUC was calculated using the bootstrap method with 1000 intervals. To compare the AUCs of different models, the DeLong testing method was applied, enabling a statistical assessment of the differences in performance metrics between the models (30). The study aims to compare the performance of radiomics features, transfer learning models, and clinical features in different models. The most effective model will then be compared to the performance of deep learning in order to identify the optimal method for screening patients for osteoporosis using chest CT scans. The Selection Criteria: Evaluating the Performance of Machine Learning Models for Osteoporosis Screening in the Test Group, Prioritizing Accuracy and AUC.

## Results

A total of 488 patients were included in the study and randomly divided into a training group and a testing group. In the training group, out of a total of 170 patients, none were diagnosed with osteoporosis, while 222 patients were identified as osteoporosis patients. Similarly, in the test group, 40 patients were found to be free from osteoporosis, while 56 patients were diagnosed with the condition. **Figures 1B–E** shows the process of feature extractionfor clinical models, radiomics models, and 2D/3D transfer learning models, respectively

### Screening of risk factors for osteoporosis

**Figure 2:**
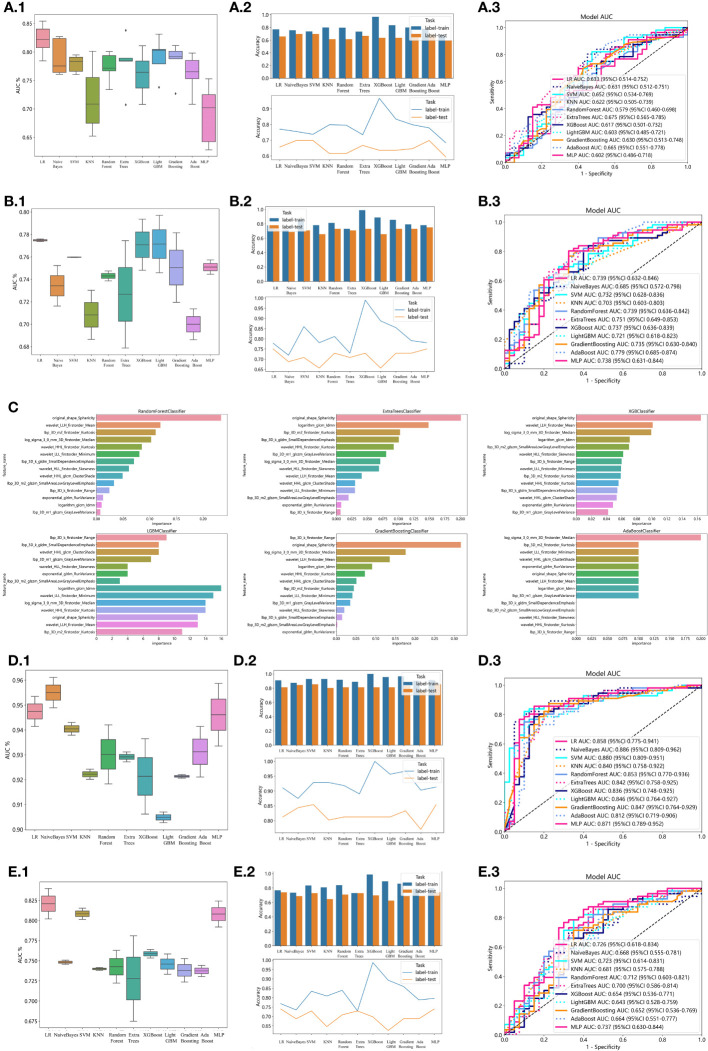
Effectiveness of radiomics models, clinical models, 2D transfer learning models, and 3D transfer models. **(A)** Effectiveness of clinical models. **(A.1)** Utilizing Machine Learning Models for Osteoporosis Screening Based on Clinical Features. **(A.2)** Assessing the Accuracy of Machine Learning Models for Osteoporosis Screening using Clinical Features. **(A.3)** Evaluation of Machine Learning Models in the Testing Group, Leveraging Clinical Features for Osteoporosis Screening: AUC and Sensitivity Analysis. **(B)** Effectiveness of radiomics models. **(B.1)** Utilizing Machine Learning Models for Osteoporosis Screening Based on Radiomics Features. **(B.2)** Assessing the Accuracy of Machine Learning Models for Osteoporosis Screening using Radiomics Features. **(B.3)** Evaluation of Machine Learning Models in the Testing Group, Leveraging Radiomics Features for Osteoporosis Screening: AUC and Sensitivity Analysis. **(C)** Weights of radiomics features in tree models. **(D)** Effectiveness of 2D Transfer Learning Model. **(D.1)** Utilizing Machine Learning Models for Osteoporosis Screening Based on 2D Transfer Learning. **(D.2)** Assessing the Accuracy of Machine Learning Models for Osteoporosis Screening using 2D Transfer Learning. **(D.3)** Evaluation of Machine Learning Models in the Testing Group, Leveraging 2D Transfer Learning for Osteoporosis Screening: AUC and Sensitivity Analysis. **(E)** Effectiveness of 3D Transfer Learning Model. **(E.1)** Utilizing Machine Learning Models for Osteoporosis Screening Based on 3D Transfer Learning. **(E.2)** Assessing the Accuracy of Machine Learning Models for Osteoporosis Screening using 3D Transfer Learning. **(E.3)** Evaluation of Machine Learning Models in the Testing Group, Leveraging 3D Transfer Learning for Osteoporosis Screening: AUC and Sensitivity Analysis.

In the univariate analysis, the p-values of gender, age, vitamin D, TPINP, and β-cross, were found to be less than 0.05. These indicators were subsequently chosen for the multivariate analysis. In the multivariate analysis, the p-values of indicators such as gender, age, and β-cross also remained below the 0.05 threshold. Based on these results, these indicators were selected as the foundation for establishing clinical models. The outcomes of both single factor analysis and multivariable analysis are presented in **Table 2**. **Table 3** and **Figure 2A** displays the performance of these features in the machine learning models. In the testing group, the AdaBoost model exhibited the highest performance. The accuracy of this model is 0.698, and the AUC is 0.665.

Table 2Screening of risk factors for osteoporosis and establishment of clinical models.

**Table:** 

Univariate analysis	Log(OR)	lower 95%CI	upper 95%CI	OR	OR lower 95%CI	OR upper 95%CI	p_value
**gender**	0.017	0.014	0.020	1.017	1.014	1.020	0.000
**age**	-0.006	-0.010	-0.002	0.994	0.990	0.998	0.007
**vitamin D**	0.001	0.000	0.002	1.001	1.000	1.002	0.018
**TPINP**	0.299	0.204	0.394	1.348	1.226	1.483	0.000
**Bcross**	0.178	0.097	0.259	1.195	1.102	1.296	0.000

**Table:** 

Multivariate analysis	Log(OR)	lower 95%CI	upper 95%CI	OR	OR lower 95%CI	OR upper 95%CI	p_value
**vitamin D**	0.000	-0.003	0.004	1.000	0.997	1.004	0.926
**TPINP**	-0.000	-0.001	0.001	1.000	0.999	1.001	0.673
**age**	0.016	0.013	0.019	1.016	1.013	1.019	0.000
**gender**	0.169	0.094	0.244	1.184	1.099	1.276	0.000
**Bcross**	0.242	0.140	0.344	1.274	1.150	1.411	0.000

TPINP, Total type I collagen amino terminal extender peptide.

β-CTX, β- Cross Laps.

**Table 3:** Effectiveness of clinical model.

Model	Accuracy	AUC	95% CI	Sensitivity	Specificity	PPV	NPV	Precision	Recall	F1	Threshold	Task
LR	0.770	0.829	0.7884 - 0.8688	0.721	0.835	0.851	0.696	0.851	0.721	0.780	0.590	Train
LR	0.656	0.633	0.5145 - 0.7516	0.696	0.600	0.709	0.585	0.709	0.696	0.703	0.465	Test
NaiveBayes	0.755	0.800	0.7549 - 0.8443	0.725	0.794	0.821	0.689	0.821	0.725	0.770	0.463	Train
NaiveBayes	0.698	0.631	0.5118 - 0.7507	0.804	0.550	0.714	0.667	0.714	0.804	0.756	0.257	Test
SVM	0.737	0.801	0.7581 - 0.8442	0.658	0.841	0.844	0.653	0.844	0.658	0.739	0.627	Train
SVM	0.698	0.652	0.5342 - 0.7694	0.821	0.525	0.708	0.677	0.708	0.821	0.760	0.354	Test
KNN	0.798	0.874	0.8416 - 0.9064	0.811	0.782	0.829	0.760	0.829	0.811	0.820	0.600	Train
KNN	0.615	0.622	0.5055 - 0.7387	0.571	0.730	0.711	0.529	0.711	0.571	0.634	0.600	Test
RandomForest	0.796	0.853	0.8155 - 0.8905	0.793	0.800	0.838	0.747	0.838	0.793	0.815	0.534	Train
RandomForest	0.615	0.579	0.4595 - 0.6985	0.696	0.500	0.661	0.541	0.661	0.696	0.678	0.467	Test
ExtraTrees	0.735	0.815	0.7737 - 0.8567	0.644	0.853	0.851	0.647	0.851	0.644	0.733	0.598	Train
ExtraTrees	0.667	0.675	0.5650 - 0.7845	0.714	0.600	0.714	0.600	0.714	0.714	0.714	0.524	Test
XGBoost	0.967	0.992	0.9858 - 0.9977	0.968	0.965	0.973	0.959	0.973	0.968	0.971	0.488	Train
XGBoost	0.635	0.617	0.5012 - 0.7319	0.732	0.500	0.672	0.571	0.672	0.732	0.701	0.319	Test
LightGBM	0.834	0.911	0.8844 - 0.9383	0.896	0.753	0.826	0.848	0.826	0.896	0.860	0.487	Train
LightGBM	0.635	0.603	0.4854 - 0.7208	0.732	0.513	0.672	0.571	0.672	0.732	0.701	0.466	Test
GradientBoosting	0.798	0.884	0.8515 - 0.9156	0.770	0.835	0.859	0.736	0.859	0.770	0.812	0.532	Train
GradientBoosting	0.646	0.630	0.5131 - 0.7477	0.696	0.605	0.696	0.575	0.696	0.696	0.696	0.447	Test
AdaBoost	0.778	0.857	0.8208 - 0.8932	0.748	0.818	0.843	0.713	0.843	0.748	0.792	0.502	Train
AdaBoost	0.698	0.665	0.5513 - 0.7777	0.875	0.450	0.690	0.720	0.690	0.875	0.772	0.472	Test
MLP	0.684	0.736	0.6873 - 0.7844	0.613	0.776	0.782	0.606	0.782	0.613	0.687	0.688	Train
MLP	0.594	0.602	0.4860 - 0.7185	0.411	0.850	0.793	0.507	0.793	0.411	0.541	0.717	Test

### Establishment of radiomics model

1834 radiomics features were extracted for each patient. Finally, 14 features were ultimately included in the study. **Table 4** and **Figure 2B** displays the performance of these radiomics features in the machine learning models. **Figure 2C** shows the importance ranking of filtered radiomics features among six tree models. In the testing group, the LR model showcased the best performance. The accuracy of this model in the test group is 0.750, and the AUC is 0.739.

**Table 4:** Effectiveness of radiomics model.

Model	Accuracy	AUC	95% CI	Sensitivity	Specificity	PPV	NPV	Precision	Recall	F1	Threshold	Task
LR	0.778	0.830	0.7896 - 0.8701	0.860	0.671	0.773	0.786	0.773	0.860	0.814	0.451	Train
LR	0.750	0.739	0.6321 - 0.8456	0.804	0.675	0.776	0.711	0.776	0.804	0.789	0.527	Test
NaiveBayes	0.719	0.796	0.7518 - 0.8393	0.644	0.822	0.822	0.638	0.822	0.644	0.722	0.562	Train
NaiveBayes	0.688	0.685	0.5717 - 0.7979	0.804	0.525	0.703	0.656	0.703	0.804	0.750	0.339	Test
SVM	0.860	0.916	0.8876 - 0.9438	0.914	0.788	0.849	0.876	0.849	0.914	0.881	0.487	Train
SVM	0.708	0.732	0.6279 - 0.8355	0.696	0.725	0.780	0.630	0.780	0.696	0.736	0.646	Test
KNN	0.781	0.860	0.8255 - 0.8938	0.820	0.729	0.798	0.756	0.798	0.820	0.809	0.600	Train
KNN	0.656	0.703	0.6029 - 0.8034	0.714	0.575	0.702	0.590	0.702	0.714	0.708	0.600	Test
RandomForest	0.811	0.870	0.8342 - 0.9053	0.856	0.753	0.819	0.800	0.819	0.856	0.837	0.496	Train
RandomForest	0.729	0.739	0.6362 - 0.8415	0.821	0.600	0.742	0.706	0.742	0.821	0.780	0.483	Test
ExtraTrees	0.730	0.792	0.7476 - 0.8355	0.860	0.559	0.718	0.754	0.718	0.860	0.783	0.532	Train
ExtraTrees	0.708	0.751	0.6491 - 0.8527	0.661	0.775	0.804	0.620	0.804	0.661	0.725	0.574	Test
XGBoost	0.990	0.999	0.9987 - 1.0000	0.986	0.994	0.995	0.983	0.995	0.986	0.991	0.637	Train
XGBoost	0.729	0.737	0.6363 - 0.8387	0.839	0.590	0.734	0.719	0.734	0.839	0.783	0.472	Test
LightGBM	0.888	0.953	0.9342 - 0.9720	0.905	0.865	0.897	0.875	0.897	0.905	0.901	0.552	Train
LightGBM	0.656	0.721	0.6180 - 0.8231	0.536	0.825	0.811	0.559	0.811	0.536	0.645	0.630	Test
GradientBoosting	0.855	0.914	0.8858 - 0.9430	0.838	0.876	0.899	0.805	0.899	0.838	0.867	0.558	Train
GradientBoosting	0.729	0.735	0.6295 - 0.8401	0.768	0.675	0.768	0.675	0.768	0.768	0.768	0.547	Test
AdaBoost	0.791	0.854	0.8177 - 0.8910	0.842	0.724	0.799	0.778	0.799	0.842	0.820	0.503	Train
AdaBoost	0.729	0.779	0.6847 - 0.8742	0.786	0.650	0.759	0.684	0.759	0.786	0.772	0.503	Test
MLP	0.781	0.846	0.8083 - 0.8837	0.842	0.700	0.786	0.773	0.786	0.842	0.813	0.506	Train
MLP	0.750	0.738	0.6314 - 0.8444	0.804	0.675	0.776	0.711	0.776	0.804	0.789	0.544	Test

### Efficiency of 2D deep learning models

**Figure 3:**
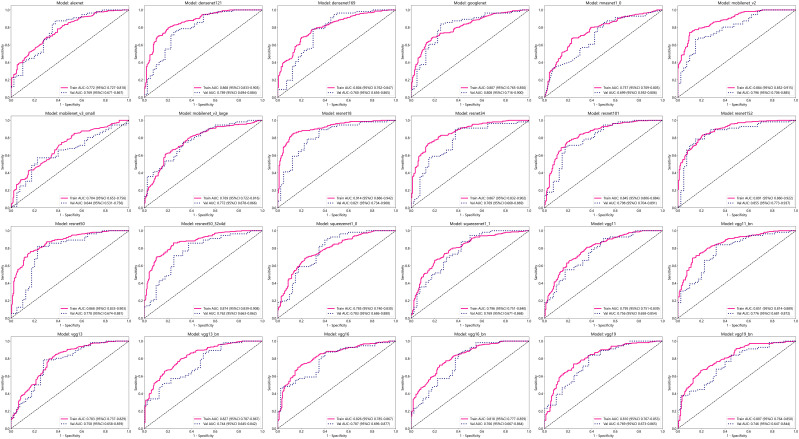
Effectiveness of 2D deep learning models.

**Figure 4:**
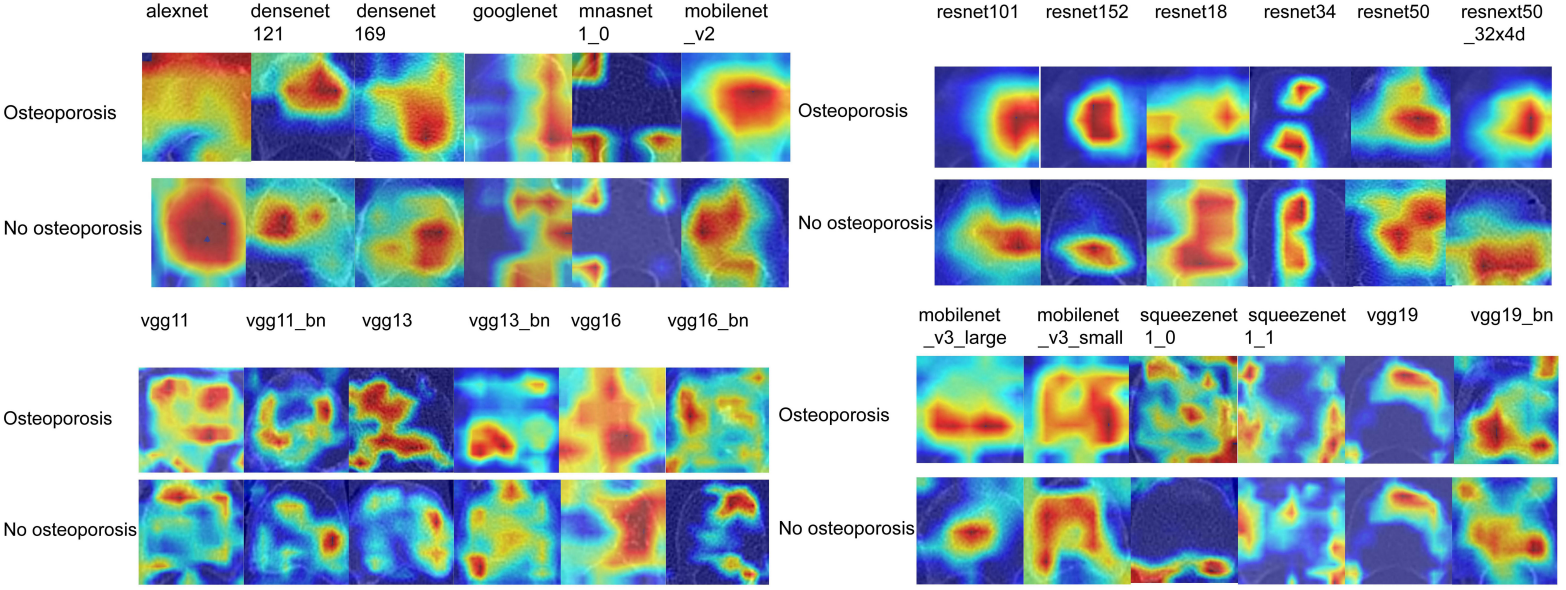
Visualization of 2D deep learning models.

After performing image processing and inputting the data, a total of 24 2D deep learning models were employed to detect osteoporosis using the maximum cross-sectional area of the ROI in chest CT scans. These findings are summarized in **Table 5** and visually presented in **Figure 3**. Additionally, the visualization results of the model can be observed in **Figure 4**. Among the various models tested for screening osteoporosis through chest CT, ResNet152 exhibited the most optimal performance. The accuracy of this model in the test group is 0.812, and the AUC is 0.855.

**Table 5:** Effectiveness of 2D deep learning models.

ModelName	Accuracy	AUC	95% CI	Sensitivity	Specificity	PPV	NPV	Precision	Recall	F1	Threshold	Cohort
alexnet	0.707	0.772	0.7268–0.8179	0.784	0.606	0.722	0.682	0.722	0.784	0.752	0.483	Train
alexnet	0.771	0.769	0.6708–0.8671	0.875	0.625	0.766	0.781	0.766	0.875	0.817	0.486	Test
densenet121	0.796	0.868	0.8330–0.9033	0.793	0.800	0.838	0.747	0.838	0.793	0.815	0.554	Train
densenet121	0.750	0.789	0.6941–0.8844	0.750	0.750	0.808	0.682	0.808	0.750	0.778	0.836	Test
densenet169	0.747	0.804	0.7618–0.8470	0.775	0.712	0.778	0.708	0.778	0.775	0.777	0.518	Train
densenet169	0.750	0.760	0.6561–0.8649	0.786	0.700	0.786	0.700	0.786	0.786	0.786	0.585	Test
googlenet	0.742	0.807	0.7650–0.8496	0.680	0.824	0.834	0.664	0.834	0.680	0.749	0.607	Train
googlenet	0.802	0.808	0.7161–0.9004	0.839	0.750	0.825	0.769	0.825	0.839	0.832	0.498	Test
mnasnet1_0	0.714	0.757	0.7095–0.8053	0.671	0.789	0.793	0.642	0.793	0.671	0.727	0.705	Train
mnasnet1_0	0.708	0.699	0.5921–0.8057	0.857	0.541	0.706	0.714	0.706	0.857	0.774	0.880	Test
mobilenet_v2	0.811	0.884	0.8517–0.9154	0.743	0.900	0.907	0.729	0.907	0.743	0.817	0.646	Train
mobilenet_v2	0.740	0.796	0.7064–0.8851	0.661	0.850	0.860	0.642	0.860	0.661	0.747	0.741	Test
mobilenet_v3_large	0.704	0.769	0.7217–0.8161	0.617	0.818	0.815	0.621	0.815	0.617	0.703	0.593	Train
mobilenet_v3_large	0.719	0.772	0.6775–0.8658	0.750	0.675	0.764	0.659	0.764	0.750	0.757	0.506	Test
mobilenet_v3_small	0.671	0.704	0.6527–0.7556	0.748	0.571	0.695	0.634	0.695	0.748	0.720	0.545	Train
mobilenet_v3_small	0.656	0.644	0.5311–0.7560	0.571	0.775	0.780	0.564	0.780	0.571	0.660	0.637	Test
resnet101	0.776	0.845	0.8061–0.8844	0.748	0.812	0.838	0.711	0.838	0.748	0.790	0.571	Train
resnet101	0.750	0.798	0.7044–0.8911	0.696	0.825	0.848	0.660	0.848	0.696	0.765	0.873	Test
resnet152	0.814	0.891	0.8601–0.9217	0.829	0.794	0.840	0.780	0.840	0.829	0.834	0.535	Train
resnet152	0.812	0.855	0.7726–0.9368	0.786	0.850	0.880	0.739	0.880	0.786	0.830	0.649	Test
resnet18	0.860	0.914	0.8855–0.9416	0.851	0.871	0.896	0.818	0.896	0.851	0.873	0.555	Train
resnet18	0.792	0.821	0.7339–0.9080	0.893	0.650	0.781	0.812	0.781	0.893	0.833	0.484	Test
resnet34	0.786	0.867	0.8320–0.9019	0.730	0.859	0.871	0.709	0.871	0.730	0.794	0.617	Train
resnet34	0.781	0.769	0.6684–0.8687	0.893	0.625	0.769	0.806	0.769	0.893	0.826	0.501	Test
resnet50	0.793	0.868	0.8329–0.9032	0.761	0.835	0.858	0.728	0.858	0.761	0.807	0.624	Train
resnet50	0.802	0.778	0.6743–0.8815	0.821	0.775	0.836	0.756	0.836	0.821	0.829	0.615	Test
resnext50_32x4d	0.814	0.874	0.8395–0.9075	0.860	0.753	0.820	0.805	0.820	0.860	0.840	0.445	Train
resnext50_32x4d	0.760	0.762	0.6627–0.8623	0.857	0.625	0.762	0.758	0.762	0.857	0.807	0.508	Test
squeezenet1_0	0.722	0.785	0.7401–0.8299	0.685	0.771	0.796	0.652	0.796	0.685	0.736	0.595	Train
squeezenet1_0	0.771	0.783	0.6861–0.8800	0.893	0.600	0.758	0.800	0.758	0.893	0.820	0.532	Test
squeezenet1_1	0.722	0.796	0.7511–0.8400	0.662	0.800	0.812	0.645	0.812	0.662	0.730	0.600	Train
squeezenet1_1	0.750	0.769	0.6706–0.8678	0.875	0.575	0.742	0.767	0.742	0.875	0.803	0.579	Test
vgg11	0.737	0.795	0.7512–0.8394	0.811	0.641	0.747	0.722	0.747	0.811	0.778	0.537	Train
vgg11	0.740	0.756	0.6578–0.8542	0.911	0.500	0.718	0.800	0.718	0.911	0.803	0.502	Test
vgg11_bn	0.768	0.851	0.8143–0.8886	0.685	0.876	0.879	0.680	0.879	0.685	0.770	0.650	Train
vgg11_bn	0.771	0.776	0.6811–0.8716	0.839	0.675	0.783	0.750	0.783	0.839	0.810	0.494	Test
vgg13	0.745	0.783	0.7370–0.8294	0.797	0.676	0.763	0.719	0.763	0.797	0.780	0.543	Train
vgg13	0.760	0.758	0.6579–0.8586	0.786	0.725	0.800	0.707	0.800	0.786	0.793	0.463	Test
vgg13_bn	0.750	0.827	0.7871–0.8669	0.793	0.694	0.772	0.720	0.772	0.793	0.782	0.549	Train
vgg13_bn	0.708	0.744	0.6454–0.8417	0.857	0.500	0.706	0.714	0.706	0.857	0.774	0.503	Test
vgg16	0.760	0.826	0.7853–0.8671	0.730	0.800	0.827	0.694	0.827	0.730	0.775	0.581	Train
vgg16	0.760	0.787	0.6958–0.8775	0.875	0.600	0.754	0.774	0.754	0.875	0.810	0.465	Test
vgg16_bn	0.740	0.818	0.7774–0.8592	0.725	0.759	0.797	0.679	0.797	0.725	0.759	0.590	Train
vgg16_bn	0.750	0.766	0.6669–0.8643	0.857	0.600	0.750	0.750	0.750	0.857	0.800	0.618	Test
vgg19	0.745	0.810	0.7675–0.8533	0.739	0.753	0.796	0.688	0.796	0.739	0.766	0.586	Train
vgg19	0.750	0.769	0.6733–0.8651	0.839	0.625	0.758	0.735	0.758	0.839	0.797	0.573	Test
vgg19_bn	0.724	0.807	0.7642–0.8497	0.662	0.806	0.817	0.646	0.817	0.662	0.731	0.578	Train
vgg19_bn	0.719	0.746	0.6475–0.8436	0.839	0.550	0.723	0.710	0.723	0.839	0.777	0.630	Test

### Efficiency of 3D deep learning models

**Figure 5:**
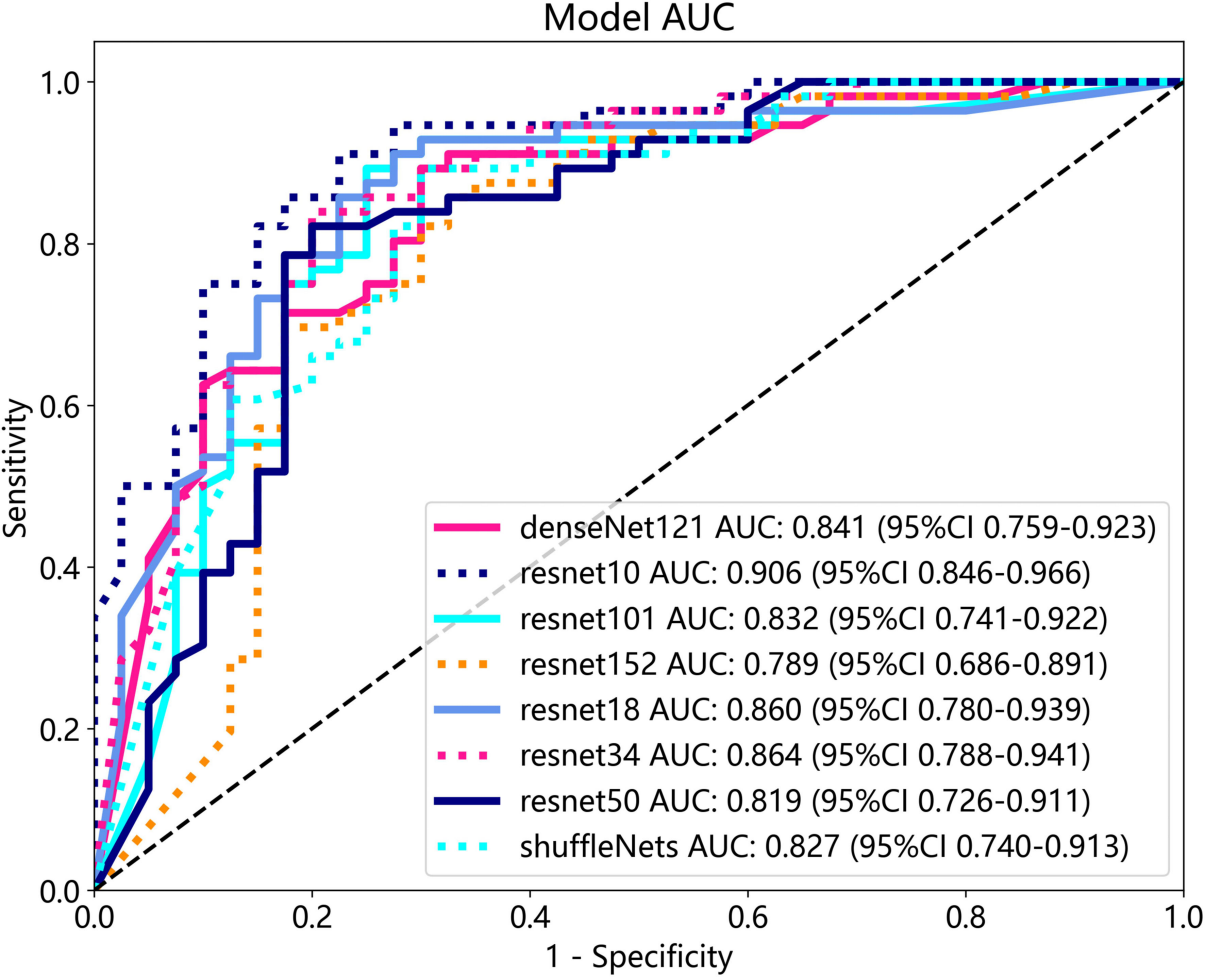
Effectiveness of 3D deep learning models in test group.

After performing image processing and inputting the data, a total of 8 3D deep learning models were employed to detect osteoporosis using the all regions of the region of interest (ROI) in chest CT scans. These findings are summarized in **Table 6** and visually presented in **Figure 5**. Among the various models tested for screening osteoporosis through chest CT, ResNet10 exhibited the most optimal performance. The accuracy of this model in the test group is 0.854, and the AUC is 0.906.

**Table 6:** Effectiveness of 3D deep learning models.

ModelName	Accuracy	AUC	95% CI	Sensitivity	Specificity	PPV	NPV	Precision	Recall	F1	Threshold	Cohort
DenseNet121	0.980	0.995	0.9885–1.0000	0.977	0.982	0.986	0.971	0.986	0.977	0.982	0.607	Train
DenseNet121	0.812	0.841	0.7591–0.9226	0.893	0.737	0.806	0.824	0.806	0.893	0.847	0.638	Test
resnet10	0.985	0.998	0.9957–1.0000	0.982	0.988	0.991	0.977	0.991	0.982	0.986	0.586	Train
resnet10	0.854	0.906	0.8456–0.9656	0.911	0.775	0.850	0.861	0.850	0.911	0.879	0.456	Test
resnet101	0.985	0.998	0.9968–1.0000	0.982	0.988	0.991	0.977	0.991	0.982	0.986	0.607	Train
resnet101	0.833	0.832	0.7413–0.9221	0.893	0.789	0.833	0.833	0.833	0.893	0.862	0.700	Test
resnet152	0.982	0.997	0.9933–0.9998	0.982	0.982	0.986	0.977	0.986	0.982	0.984	0.564	Train
resnet152	0.781	0.789	0.6863–0.8909	0.857	0.771	0.787	0.771	0.787	0.857	0.821	0.367	Test
resnet18	0.967	0.991	0.9843–0.9985	0.959	0.976	0.982	0.949	0.982	0.959	0.970	0.667	Train
resnet18	0.833	0.860	0.7803–0.9389	0.911	0.744	0.823	0.853	0.823	0.911	0.864	0.763	Test
resnet34	0.926	0.971	0.9554–0.9865	0.932	0.918	0.937	0.912	0.937	0.932	0.935	0.619	Train
resnet34	0.823	0.864	0.7879–0.9407	0.839	0.821	0.855	0.780	0.855	0.839	0.847	0.872	Test
resnet50	0.977	0.996	0.9923–0.9997	0.977	0.976	0.982	0.971	0.982	0.977	0.980	0.686	Train
resnet50	0.812	0.819	0.7261–0.9114	0.821	0.842	0.852	0.762	0.852	0.821	0.836	0.580	Test
ShuffleNet	0.990	0.999	0.9985–1.0000	0.991	0.988	0.991	0.988	0.991	0.991	0.991	0.454	Train
ShuffleNet	0.812	0.827	0.7404–0.9127	0.893	0.757	0.806	0.824	0.806	0.893	0.847	0.506	Test

### Extraction and efficiency of 2D transfer learning

ResNet152, the most potent model in the arena of 2D deep learning, was identified as the top choice for feature extraction in the domain of 2D deep transfer learning based on the results of the previous step. In the testing group, the SVM model showcased the best performance. The accuracy of this model in the test group is 0.854, and the AUC is 0.880. **Table 7** and **Figure 2D** displays the performance of these features.

**Table 7:** Effectiveness of 2D transfer learning.

Model	Accuracy	AUC	95% CI	Sensitivity	Specificity	PPV	NPV	Precision	Recall	F1	Threshold	Task
LR	0.911	0.966	0.9513 - 0.9816	0.932	0.882	0.912	0.909	0.912	0.932	0.922	0.475	Train
LR	0.812	0.858	0.7748 - 0.9413	0.732	0.925	0.932	0.712	0.932	0.732	0.820	0.812	Test
NaiveBayes	0.875	0.957	0.9393 - 0.9741	0.856	0.900	0.918	0.827	0.918	0.856	0.886	0.856	Train
NaiveBayes	0.844	0.886	0.8092 - 0.9622	0.804	0.947	0.918	0.766	0.918	0.804	0.857	0.960	Test
SVM	0.929	0.975	0.9608 - 0.9888	0.946	0.906	0.929	0.928	0.929	0.946	0.937	0.500	Train
SVM	0.854	0.880	0.8091 - 0.9507	0.821	0.900	0.920	0.783	0.920	0.821	0.868	0.664	Test
KNN	0.929	0.974	0.9611 - 0.9865	0.950	0.900	0.925	0.933	0.925	0.950	0.938	0.600	Train
KNN	0.802	0.840	0.7576 - 0.9224	0.821	0.816	0.836	0.756	0.836	0.821	0.829	0.600	Test
RandomForest	0.918	0.972	0.9571 - 0.9865	0.919	0.918	0.936	0.897	0.936	0.919	0.927	0.584	Train
RandomForest	0.812	0.853	0.7699 - 0.9359	0.750	0.923	0.913	0.720	0.913	0.750	0.824	0.657	Test
ExtraTrees	0.890	0.946	0.9245 - 0.9669	0.937	0.829	0.878	0.910	0.878	0.937	0.906	0.525	Train
ExtraTrees	0.812	0.842	0.7581 - 0.9254	0.821	0.800	0.852	0.762	0.852	0.821	0.836	0.554	Test
XGBoost	1.000	1.000	1.0000 - 1.0000	1.000	1.000	1.000	1.000	1.000	1.000	1.000	0.597	Train
XGBoost	0.812	0.836	0.7477 - 0.9246	0.786	0.895	0.880	0.739	0.880	0.786	0.830	0.656	Test
LightGBM	0.957	0.990	0.9826 - 0.9965	0.995	0.906	0.932	0.994	0.932	0.995	0.963	0.504	Train
LightGBM	0.812	0.846	0.7636 - 0.9275	0.768	0.875	0.896	0.729	0.896	0.768	0.827	0.651	Test
GradientBoosting	0.967	0.991	0.9829 - 0.9983	0.977	0.953	0.964	0.970	0.964	0.977	0.971	0.550	Train
GradientBoosting	0.833	0.847	0.7643 - 0.9294	0.875	0.775	0.845	0.816	0.845	0.875	0.860	0.500	Test
AdaBoost	0.903	0.971	0.9578 - 0.9834	0.919	0.882	0.911	0.893	0.911	0.919	0.915	0.490	Train
AdaBoost	0.771	0.812	0.7187 - 0.9058	0.732	0.825	0.854	0.687	0.854	0.732	0.788	0.535	Test
MLP	0.913	0.963	0.9458 - 0.9792	0.964	0.847	0.892	0.947	0.892	0.964	0.926	0.407	Train
MLP	0.854	0.871	0.7894 - 0.9517	0.857	0.850	0.889	0.810	0.889	0.857	0.873	0.580	Test

### Extraction and efficiency of 3D transfer learning

ResNet10, the most potent model in the arena of 3D deep learning, was identified as the top choice for feature extraction in the domain of 3D deep transfer learning based on previous research findings. In the testing group, the MLP model showcased the best performance. The accuracy of this model in the test group is 0.740, and the AUC is 0.737. **Table 8** and **Figure 2E** displays the performance of these features.

**Table 8:** Effectiveness of 3D transfer learning.

Model	Accuracy	AUC	95% CI	Sensitivity	Specificity	PPV	NPV	Precision	Recall	F1	Threshold	Task
LR	0.768	0.841	0.8017 - 0.8800	0.743	0.800	0.829	0.705	0.829	0.743	0.784	0.592	Train
LR	0.740	0.726	0.6177 - 0.8341	0.875	0.564	0.731	0.759	0.731	0.875	0.797	0.439	Test
NaiveBayes	0.735	0.779	0.7325 - 0.8258	0.788	0.665	0.754	0.706	0.754	0.788	0.771	0.379	Train
NaiveBayes	0.688	0.668	0.5546 - 0.7811	0.696	0.692	0.750	0.614	0.750	0.696	0.722	0.360	Test
SVM	0.834	0.904	0.8743 - 0.9346	0.770	0.918	0.924	0.754	0.924	0.770	0.840	0.669	Train
SVM	0.729	0.723	0.6143 - 0.8312	0.786	0.650	0.759	0.684	0.759	0.786	0.772	0.557	Test
KNN	0.809	0.894	0.8653 - 0.9233	0.824	0.788	0.836	0.775	0.836	0.824	0.830	0.600	Train
KNN	0.646	0.681	0.5751 - 0.7879	0.696	0.605	0.696	0.575	0.696	0.696	0.696	0.600	Test
RandomForest	0.839	0.888	0.8557 - 0.9203	0.923	0.729	0.817	0.879	0.817	0.923	0.867	0.493	Train
RandomForest	0.708	0.712	0.6030 - 0.8206	0.804	0.590	0.726	0.676	0.726	0.804	0.763	0.508	Test
ExtraTrees	0.730	0.811	0.7692 - 0.8527	0.676	0.800	0.815	0.654	0.815	0.676	0.739	0.574	Train
ExtraTrees	0.729	0.700	0.5856 - 0.8144	0.839	0.590	0.734	0.719	0.734	0.839	0.783	0.561	Test
XGBoost	0.987	0.999	0.9988 - 1.0000	0.977	1.000	1.000	0.971	1.000	0.977	0.989	0.614	Train
XGBoost	0.698	0.654	0.5357 - 0.7715	0.857	0.475	0.696	0.704	0.696	0.857	0.768	0.343	Test
LightGBM	0.893	0.958	0.9410 - 0.9760	0.851	0.947	0.955	0.830	0.955	0.851	0.900	0.597	Train
LightGBM	0.625	0.643	0.5280 - 0.7587	0.589	0.675	0.717	0.540	0.717	0.589	0.647	0.588	Test
GradientBoosting	0.860	0.937	0.9132 - 0.9601	0.802	0.935	0.942	0.783	0.942	0.802	0.866	0.609	Train
GradientBoosting	0.688	0.652	0.5357 - 0.7688	0.714	0.650	0.741	0.619	0.741	0.714	0.727	0.564	Test
AdaBoost	0.788	0.844	0.8051 - 0.8834	0.874	0.676	0.779	0.804	0.779	0.874	0.824	0.497	Train
AdaBoost	0.688	0.664	0.5509 - 0.7768	0.821	0.500	0.697	0.667	0.697	0.821	0.754	0.483	Test
MLP	0.796	0.858	0.8205 - 0.8951	0.851	0.724	0.801	0.788	0.801	0.851	0.825	0.495	Train
MLP	0.740	0.737	0.6304 - 0.8437	0.768	0.700	0.782	0.683	0.782	0.768	0.775	0.548	Test

### Comparison of the effectiveness of screening osteoporosis

**Table 9** presents the comparison of the effectiveness of various features in screening for osteoporosis among machine learning models. In the LR and AdaBoost models, the radiomics features were found to be more effective in screening for osteoporosis compared to clinical features. However, in the other models, there was no statistically significant difference between the effectiveness of the two feature types. On the other hand, the effectiveness of 3D transfer learning model was not superior to clinical and radiomics features in any of the models. Furthermore, among all the models, the 2D transfer learning model were superior to clinical features in screening for osteoporosis. Additionally, the effectiveness of 2D transfer learning was found to be superior to 3D transfer learning in all models. Moreover, when considering the seven models (LR, NaiveBayes, SVM, KNN, LightGBM, GradientBoosting, and MLP), the effectiveness of 2D transfer learning model in screening for osteoporosis was superior to radiomics features.

**Table 9:** Comparison of the effectiveness of screening osteoporosis through clinical models and radiomics, 2D deep learning features, and 3D deep learning features by Delong test.

Model	Clinical VS Radiomics	Clinical VS 2D Transfer Learning	Clinical VS 3D Transfer Learning	2D Transfer Learning VS 3D Transfer Learning	Radiomics VS 2D Transfer Learning	Radiomics VS 3D Transfer Learning	Task
LR	0.79	<0.01	0.52	<0.01	<0.01	0.70	Train
LR	0.04	<0.01	0.13	0.02	0.04	0.82	Test
NaiveBayes	0.91	<0.01	0.64	<0.01	<0.01	0.57	Train
NaiveBayes	0.39	<0.01	0.62	<0.01	<0.01	0.80	Test
SVM	<0.01	<0.01	<0.01	<0.01	<0.01	0.47	Train
SVM	0.08	<0.01	0.30	0.01	<0.01	0.89	Test
KNN	0.65	<0.01	0.23	<0.01	<0.01	0.11	Train
KNN	0.20	<0.01	0.48	0.01	0.02	0.77	Test
RandomForest	0.36	<0.01	0.07	<0.01	<0.01	0.35	Train
RandomForest	<0.01	<0.01	0.05	0.03	0.05	0.63	Test
ExtraTrees	0.41	<0.01	0.85	<0.01	<0.01	0.52	Train
ExtraTrees	0.17	<0.01	0.70	0.02	0.12	0.40	Test
XGBoost	0.01	<0.01	1.00	0.12	0.12	0.01	Train
XGBoost	0.05	<0.01	0.61	<0.01	0.09	0.24	Test
LightGBM	<0.01	<0.01	<0.01	<0.01	<0.01	0.63	Train
LightGBM	0.05	<0.01	0.57	<0.01	0.03	0.24	Test
GradientBoosting	0.09	<0.01	<0.01	<0.01	<0.01	0.12	Train
GradientBoosting	0.09	<0.01	0.76	<0.01	<0.01	0.22	Test
AdaBoost	0.98	<0.01	0.66	<0.01	<0.01	0.63	Train
AdaBoost	0.04	0.02	0.99	0.02	0.54	0.06	Test
MLP	<0.01	<0.01	<0.01	<0.01	<0.01	0.71	Train
MLP	0.06	<0.01	0.07	0.02	0.02	0.98	Test

### Assessing the effectiveness of the optimal machine learning model and deep learning technology for osteoporosis screening

The optimal machine models for screening osteoporosis based on each feature were chosen as the reference for comparison with deep learning techniques. When utilizing clinical features for osteoporosis screening, the AdaBoost model demonstrates the highest performance. The LR model, on the other hand, shows the best performance when employing radiomics features for osteoporosis screening. For 2D transfer learning, the SVM model exhibits the most optimal performance, while for 3D transfer learning, the MLP model shows the highest performance. Among the various 2D deeplearning models, ResNet152 exhibited the most optimal performance. Among the various 3D deeplearning models, ResNet10 exhibited the most optimal performance. The comparison between these models is presented in **Table 10**. In the test group, the AUC (Area Under the Curve) did not show any significant difference between 2D deep learning and 3D deep learning methods. However, when compared to clinical models, radiomics models, and 3D transfer learning models, the AUC of 3D deep learning was significantly better. Interestingly, there was no statistical difference in AUC when comparing 3D deep learning with 2D transfer learning models. On the other hand, the AUC of 2D deep learning was superior to clinical models, but there was no statistically significant difference between the AUC of 2D deep learning and radiomics models or 2D transfer learning models or 3D transfer learning models.These results are presented in **Table 10**.

**Table 10:** Comparison of the optimal machine learning model and deep learning technology for osteoporosis screening by delong test.

2D Deep Learning VS Clinical(AdaBoost)	2D Deep Learning VS Radiomics(LR)	2D Deep Learning VS 2D Transfer Learning(SVM)	2D Deep Learning VS 3D Transfer Learning(MLP)	2D Deep Learning VS 3D Deep Learning	Task
0.11	0.01	<0.01	0.18	<0.01	Train
<0.01	0.06	0.31	0.05	0.26	Test
3D Deep Learning VS Clinical(AdaBoost)	3D Deep Learning VS Radiomics(LR)	3D Deep Learning VS 2D Transfer Learning(SVM)	3D Deep Learning VS 3D Transfer Learning(MLP)		Task
<0.01	<0.01	<0.01	<0.01		Train
<0.01	<0.01	0.55	<0.01		Test

## Discussion

Our study provides initial evidence supporting the potential of using chest CT for osteoporosis screening. Moreover, we observed that deep learning technology, and transfer learning technology based on chest CT are more effective than bone transition biomarkers for screening osteoporosis. Typically, in a tertiary hospital in China, the cost of a chest CT examination is around $26, while a DXA examination costs approximately $23. On the other hand, a bone turnover marker examination is priced at around $48. Osteoporosis is a silent and widespread condition, making screening crucial for identifying potential patients early on (31). Our findings suggest that conducting bone turnover biomarker testing solely for the purpose of osteoporosis screening may not be necessary. On the other hand, chest CT scans serve multiple purposes such as lung tumor screening and exclusion of pneumonia (32). Elderly individuals and the female demographic, with a particular emphasis on Asian women, are disproportionately susceptible to lung cancer. Consequently, some experts advocate for the inclusion of chest CT scans as part of routine health screenings for these groups (33). Interestingly, our study also found a correlation between age, gender, and osteoporosis, which coincides with the population commonly advised to undergo regular chest CT examinations. Older age and female gender have been consistently identified as risk factors for osteoporosis in various studies (34). Specifically, postmenopausal women in the older age group are considered a high-risk population for this condition (35). Regular chest CT examinations are often recommended for individuals in this group (36). As DXA screening for osteoporosis has not been widely adopted due to limited awareness regarding the risks associated with osteoporosis (37), utilizing chest CT for osteoporosis screening can not only benefit potential patients but also help save a substantial amount of money for medical insurance funds. By combining osteoporosis screening with routine chest CT scans, we can effectively identify at-risk individuals and allocate resources more efficiently.

There are various reasons why bone turnover markers cannot be used for osteoporosis screening. In our study, we examined three different BTM as research subjects, which were vitamin D, TPINP, and β- Cross. Initially, in the univariate regression analysis, all three markers were found to have associations with the occurrence of osteoporosis. However, in the subsequent multivariable analysis, it was determined that only β- Cross showed a significant relationship with the occurrence of osteoporosis, along with the variables of age and gender. Sufficient levels of vitamin D have been shown to enhance the absorption of calcium and facilitate the process of bone mineralization (38). Vitamin D deficiency is a prevalent issue that warrants attention, and it is not limited to individuals with osteoporosis (39). Furthermore, many osteoporosis patients are already receiving vitamin D supplementation as part of their treatment, which can elevate their blood levels of vitamin D. Consequently, this can potentially hinder the diagnostic effectiveness of using vitamin D as a marker for osteoporosis. TPINP primarily indicates bone metabolism and can be utilized to assess the effectiveness of anti-osteoporosis treatments (40). It is important to note that osteoporosis can arise from both bone metabolism abnormalities and bone loss (41). However, not all individuals with osteoporosis will exhibit significant disruptions in bone metabolism. This might explain why TPINP cannot always accurately determine the presence of osteoporosis in patients. β- Cross is a marker that indicates the level of bone resorption activity by osteoclasts (42). It is widely recognized as the most effective bone turnover marker for identifying the presence of osteoporosis in patients (43). In our research, β- Cross is believed to indicate the occurrence of osteoporosis and constitutes a clinical model with two variables: gender and age. However, the clinical efficacy of this model is not entirely satisfactory, with an accuracy of 0.698 and an AUC of 0.665.

However, the imaging features obtained through chest CT imaging greatly improve the accuracy of identifying osteoporosis. This also provides a preliminary screening for the presence of osteoporosis for patients who undergo regular chest CT examinations. The first features to be used were extracted through radiomics methods. As a newly developed technology, computed tomography (CT) radiomics has the ability to identify radiomic features that are challenging to recognize visually. This advanced approach offers a convenient, comprehensive, and accurate method for diagnosing osteoporosis (44). In our study, the radiomics model demonstrated an accuracy of 0.750 and an AUC of 0.739 for recognizing osteoporosis, the 95% confidence interval is 0.6321–0.8456. Compared to clinical models, radiomics models have shown better potential for osteoporosis screening in some machine learning models. However, compared to other previous studies, such as using HU values on chest CT to screen for osteoporosis with accuracy and AUC of 0.831 and 0.972 (45), the effectiveness of using chest CT radiomics technology to screen for osteoporosis in this study is still unsatisfactory.

Deep learning technology has emerged as a valuable tool for the diagnosis of osteoporosis, with numerous studies demonstrating its effectiveness (46). In our study, we employed a combination of 2D and 3D deep learning models to screen for osteoporosis using chest CT scans. Specifically, we utilized 24 widely used 2D deep learning models and 8 commonly used 3D deep learning models. The effectiveness of 2D and 3D deep learning models based on chest CT scans in screening for osteoporosis is significantly improved compared to clinical models that rely on bone turnover markers. While there was no statistically significant difference in performance between 2D and 3D deep learning models in the test group, it was observed that the 3D deep learning model outperformed radiomics models in terms of performance. The method of extracting 2D transfer learning has been proven to improve the effectiveness of disease prediction (47). In our study, the 2D transfer learning model showed good performance, with an accuracy of 0.854 and an AUC of 0.880. However, it is worth noting that the 3D transfer learning model did not demonstrate a better AUC (Area Under the Curve), possibly due to the overfitting phenomenon caused by the recognition of excessive image information by the extracted 3D deep learning model (47). The development of enhanced deep learning models based on 3D medical images holds the potential to further improve this phenomenon. Although 2D transfer learning models demonstrate better AUC when compared to standard 2D deep learning models, they do not significantly outperform 3D deep learning models. Therefore, researchers suggest that both 3D deep learning technology and 2D transfer learning technology should be prioritized when utilizing chest CT scans for osteoporosis screening.

Recognizing several limitations of this study is of utmost importance. Firstly, the absence of external validation is a noteworthy concern and should be given priority in future research efforts. Secondly, it is worth noting that the ROI delineation utilized in this study employed a combination of manual and semi-automatic methods.

In conclusion, our study indicates that bone turnover markers may not be necessary for osteoporosis screening. Instead, a combination of 3D deep learning and 2D transfer learning techniques based on chest CT scans can be considered as effective alternatives for osteoporosis screening.

## Data availability statement

The datasets presented in this study can be found in online repositories. The names of the repository/repositories and accession number(s) can be found in the article/supplementary material.

## Ethics statement

The studies involving humans were approved by The Second Affiliated Hospital of Fujian Medical University. The studies were conducted in accordance with the local legislation and institutional requirements. Written informed consent for participation was not required from the participants or the participants’ legal guardians/next of kin in accordance with the national legislation and institutional requirements. Written informed consent was not obtained from the individual(s) for the publication of any potentially identifiable images or data included in this article because This is a retrospective study. After application, the ethics committee agreed to cancel informed consent, but the patient’s privacy was protected.

## Author contributions

KF: Writing – original draft. XZ: Software, Writing – original draft. XL: Data curation, Writing – review & editing. ZD: Writing – review & editing.
